# Treatment of Alcoholism

**Published:** 1879-04

**Authors:** 


### EXCERPTA.

---

Treatment of Alcoholism —Some of the most distressing cases we, as medical men, are called upon to attend are those of alcoholism, and it has, unfortunately, fallen to my lot during the last few years to have several, from time to time, under my charge. A good deal has been written by different persons with regard to treatment, but I do not think this ought to deter one from putting on record his own personal observations, since it is only by accumulation of evidence that proper conclusions can be arrived at. As far as I can see, there would appear to be three different stages in the disease, viz:

1. *Sleeplsssness*, accompanied by a hard, quick pulse; loss of appetite in the morning, and morning sickness.

2. *Drowsiness*, accompanied by a slow, somewhat compressible and excitable pulse; complete loss of appetite; and constant sickness. The blood has in it an excessive amount of hydrocarbon.

3. *Delirium*, accompanied by complete absence of sleep and the presence of horrible apparitions, especially at night. The pulse is small, quick, easily excitable and compressible. The blood is deficient in red corpuscles. Hydrocarbons are present in poisonous quantities; the brain undergoes little or no repair. The vaso-motor nerve influence is almost entirely lost. The treatment I have found beneficial in each stage is the following:

*First stage.*—Tr. rhei, . . . . min. x.
Tr. card. co., . . . ʒ ss.
Tr. hyoscyami, . . . ʒ ss.
Acid. hydrocyanic. dil., . min. iij.
Sp. chloroformi, . . . min. xv.
Aquam ad ℥j. quartis horis.

The prussic acid acts as a sedative to the stomach, heart and brain. The hyoscyamus has also, to a certain extent, the same effect.

Abstinence from stimulants in this, as in the other stages, is strictly enjoined, but when I find it difficult to get this carried out, I allow a glass of claret three times a day. It is essential that the patient gets plenty of light and easily digestible food, and with this object I order Brand's essence of beef, milk and eggs beaten up together, and barley water. This diet is suitable to each stage. The only thing to be said is the more the depression the more the nourishment.

*Second stage.*—The treatment should be the same as just described, only it is as well to omit the prussic acid, as there is not the same excitement present.

*Third stage.*—Chloral should be given in thirty-grain doses every four hours, till sleep comes on, and then repeated as often as necessary. The nouishment should be by no means forgotten, and stimulants should be strictly forbidden.

If chloral is gone on with beyond a certain time, a sleepless condition recurs, when nux vomica and gentian should be given as follows:

| | |
|---|---|
| Tr. nucis vomicæ, . . . | min. x. |
| Tr. gentian co., . . . . | ʒ ss. |
| Ess. limonis., . . . . . | min j. |
| Sp. chloroform, . . . . | min xv. |

Aquam ad ℥i. ter quaterve die.

This rarely fails to reinduce sleep, but if persisted in long after it has produced its effect, sleeplessness returns. When this is the case the tincture of gentian, calumba or chiretta should be given alone.—*Dr. Atkinson, in London Pract.*

